# Supplementary material for: Are carbon emissions trading and green financial instruments synergistic? -Comprehensive quantitative research based on content analysis
Source: PLoS One. 2024 Mar 7;19(3):e0298601. doi: 10.1371/journal.pone.0298601 (PMC10919601; doi:10.1371/journal.pone.0298601)
Supplement: S1 Data — (ZIP) [file pone.0298601.s001.zip › Data/地级市Z_F工作报告.zip!/║■─╧/│ñ╔│╩╨/└┤╫╘╣1⁄2╓┌║┼í╛┬φ┐╦╩2╛▌═°í┐_2007.docx]

各位代表：

　　现在，我代表市人民政府向大会作工作报告，请予审议，并请各位政协委员和其他列席人员提出意见。

　 一、2006年工作回顾

　　过去的一年，在市委的正确领导下，全市人民坚持用科学发展观统领经济社会发展全局，紧紧抓住国家实施中部崛起战略和建设社会主义新农村机遇，奋发进取，开拓创新，完成了市十二届人大四次会议确定的目标任务，实现了“十一五”规划的良好开局。

　　（一）加快经济增长方式转变，国民经济继续保持快速健康的发展态势。全年实现地区生产总值1780亿元，增长14.8%（预计数，下同）；万元GDP综合能耗下降4%；完成全社会固定资产投资1086亿元，增长22.2%；完成地方财政收入177.01亿元，增长27%；城乡居民人均可支配收入分别达13924元和5438元，增长12%和14.9%；非公有制经济占全市经济总量达55%。大力推进工业项目年建设，实现全部工业总产值1650.95亿元，增长26.9%。农业产业化进程加快，农产品加工总值达220亿元，增长18%。实现社会消费品零售总额866亿元，增长16.4%，我市被商务部确定为“品牌万里行”中心城市和零售企业分类定级试点城市；区域性金融中心建设正式启动，金融业带动服务经济发展的能力增强；全年入境旅游人数达40.6万人次，增幅居中西部省会城市之首，旅游总收入达222.6亿元。长沙经济开始走向速度与质量、结构与效益相协调的发展轨道。

　　（二）加强城乡建设和管理，城乡面貌发生新的变化。全年完成城建重点工程项目83个，投资114.8亿元，城市综合承载功能进一步增强。三汊矶大桥竣工通车、二环线全线贯通，“六桥三环”城市发展蓝图已经变成现实。100个社区环境综合整治如期完工。新的门前市容环境卫生责任制正式实施，我市获全国城市管理先进集体十佳标兵称号。社会主义新农村建设全面启动，百村示范和千村整治工程稳步推进，完成41个乡镇和210个村规划编制，新建改造农村公路1724公里，完成水利投入11.6亿元。小城镇建设完成投入67.3亿元，城市化水平达55.9%。国土资源保护、环境保护和治理成效明显，城市空气优良率达78.1%，9个区、县（市）生态示范区建设通过国家验收，我市成为全国第三个国家森林城市和全国水环境治理优秀范例城市。长沙创建最佳宜居城市的目标正在逐步实现。

　　（三）深入推进改革开放，城市活力与影响力显著增强。国有企业改革继续深化，国有资产监管体系进一步完善，文化体制改革稳步推进，公用事业改革基本完成，农村综合配套改革取得新进展。创新招商方式，首开以资产并购设立外商投资企业先河；“伦敦洽谈会”、首届“中部投资贸易博览会”取得实效，共引进外来资金473亿元，增长47%，实际到位外资12亿美元，增长33%，居中西部地区首位。市外贸归口管理企业完成进出口总额23亿美元，其中出口13.8亿美元，分别增长14.2%和37.1%。对外交往进一步拓展，成功举办第五届中日地方交流研讨会、国际版权合作推进会、33家外资银行负责人长沙行等重大对外交流合作活动。长沙的知名度和影响力不断提升。

　　（四）统筹经济社会协调发展，人民生活质量不断提高。全年市本级用于农业、教育、科技、社会保障和城乡公共设施建设资金达48.32亿元，增长25.9%。创新型城市建设正式启动，专利申请量达4714件，增长18%。教育“两免一补”共惠及城乡49.6万中小学生。330.3万农民参加新型农村合作医疗，参合率达84.6%；城市社区卫生服务机构覆盖率达92%。成功承办第二届湖南艺术节，湘绣、浏阳花炮入选国家非物质文化遗产名录。现代女性频道实现全国覆盖，194个20户以上自然村实现广播电视“村村通”。完成贺龙体育运动学校搬迁，成功举办首届农民运动会，获省十届运动会金牌和奖牌数第一。全面完成省、市八件实事，新增城镇就业8.72万人，新增转移农村劳动力6.02万人；建设廉租房1.36万平方米、经济适用房50.1万平方米；新建改造城区站厕248所，农村改厕1.2万户；解决农村1.3万人饮水安全；城市低保再次提标；改扩建乡镇卫生院22所、敬老院28所，千人复明工程救助1039人，社会精神病人医疗救助2.1万人次，特困家庭大病医疗救助1.34万人次，慈善资助贫困学生就读大学2014人。一个覆盖全市的社会保障体系基本形成。

　　（五）切实推进民主法制建设，创建平安长沙、文明长沙取得新的进展。自觉接受人大及其常委会的法律监督和政协的民主监督，认真听取各民主党派、人民团体和各界人士的意见和建议。全年办复人大代表议案、建议和政协委员提案、建议案576件，满意度达98%。提请市人大常委会审议和通过地方性法规4件，制定政府规章2件。顺利完成第六次社区居委会换届选举工作。机关作风建设年活动和治理商业贿赂工作效果显著，政务公开范围不断扩大，公务员廉洁从政意识、自律行为进一步增强。“五五”普法正式实施，维护社会稳定、打击各类违法犯罪取得新的成效。信访渠道更加畅通，大信访格局初步形成。严格安全生产责任制和综合监管，安全生产形势明显好转。成功举办第三届全国公民道德论坛。妇女儿童生存发展状况取得长足进步。公务员法实施和工资制度改革工作全面启动。国防教育、国防后备力量建设进一步加强。我市获全国经济普查工作先进单位、全国信访工作先进集体、全国科协工作先进集体、全省双拥模范城市、全省计生工作模范市、全省社会治安综合治理先进市称号。审计、统计、物价、人防、民族宗教、台办、质量监督、工商、税务、食品药品监督、海关、电力、市志、档案、气象、水文、地震、残联、侨联、关协、妇儿工委、老龄等工作取得新进步。平安长沙、文明长沙、和谐长沙建设得到人民群众的普遍认同、积极参与和大力支持。

　　这些成绩的取得，是省委、省政府和市委正确领导的结果，是市人大、市政协监督、支持的结果，是各位代表和政协委员和衷共济的结果，是全市人民团结奋斗的结果。在此，我代表市人民政府，向全市人民，向驻长人民解放军、武警官兵和政法干警，向各民主党派、工商联、人民团体和各界人士，向所有关心和支持长沙建设与发展的国内外朋友，表示衷心的感谢和崇高的敬意！

　　在总结成绩的同时，我们也要清醒地看到面临的困难和问题，主要是：区域竞争压力增大，长沙经济总量偏小、结构不优，特别是工业经济在中部省会城市中尚未形成比较优势；投资增长呈下降趋势，尤其是工业和生产经营性投资比重偏低；农业增产增效、农民增收和农村基础设施建设任务仍然艰巨；节能降耗治污面临严峻形势，生态环境和资源保护需加大力度；经济秩序、城市管理、社会治安、关心弱势群体、维护群众利益等方面还存在薄弱环节；政府机关作风和管理有待进一步改进，公务员的公仆、勤俭、廉洁、服务意识和行政效率必须进一步提高。这些问题，我们将在今后的工作中认真加以解决。

　 二、2007年工作总体目标和主要措施

　　2007年是全面贯彻党的十六届六中全会和省第九次、市第十一次党代会精神的第一年。全市经济社会发展的主要预期目标是：　 地区生产总值增长13%以上，突破2000亿元；全社会固定资产投资总额增长18%以上，完成1300亿元；社会消费品零售总额增长15%以上，达到1000亿元；地方财政收入增长15%以上，突破200亿元；城乡居民人均可支配收入分别增长9%和10%以上，达到15000元和6000元；城镇登记失业率控制在4.5%以内；人口自然增长率控制在5.5‰以内；单位GDP综合能耗下降4%；主要污染物排放总量下降4%。

　　各位代表，由于全市人民的共同努力和艰苦创业，经过“十五”以来的建设发展，长沙已步入了加快发展的轨道，奠定了较为坚实的发展基础，积蓄了很大的发展后劲，现在我们已站在一个新的发展起点上。全市人民实现长沙又好又快、率先发展的信心更加坚定，盼望长沙在区域竞争中不断提升综合竞争实力和对外影响力的愿望更加强烈。综观全局，我们虽然面临诸多的困难和挑战，但更面临良好的发展机遇。据国际货币基金组织预测，今年全球经济将继续保持较快增长势头；中央提出要保持和扩大良好的发展势头，将加大对重点领域和薄弱环节的支持力度；入世过渡期结束，国际和国内沿海发达地区产业转移和资本流动将进一步加速；国家实施中部崛起发展战略，明确长株潭等比照东北老工业基地改造享受相关支持政策；省委、省政府提出的科学发展、又好又快、富民强省的发展战略，并要求实施长株潭一体化和“3＋5”城市群发展的重大决策取得实质性的进展，更鼓舞和寄望长沙加快发展，增强省会的辐射带动能力，为全省经济发展作出更大贡献。因此，我们必须志存高远，顺应潮流，自觉加压，以发展振奋精神，用发展凝聚人心，靠发展战胜挑战。要乘势而上，奋力拼搏，在新的一年里使长沙经济和社会事业发展再上新台阶。为此，要着力抓好以下六个方面的工作：

　　（一）突出推进新型工业化，着力增强工业核心竞争力。推进新型工业化是实现长沙又好又快发展的第一推动力。一要加快百亿元级产业集群发展，优化工业经济布局。围绕做大做强工程机械、汽车等优势产业集群，重点培育和壮大长沙卷烟、长丰集团、北汽福田、中联重科、三一重工等核心支柱企业，大力发展中小型配套企业，加强生产性服务业与现代工业的融合。扶植壮大电子信息、新材料、生物医药等新兴产业，迅速实现产业化和规模化。运用现代信息技术改造提升烟花、食品等优势传统产业。优化园区和产业布局，健全园区管理体制，构建以高新区、经开区为核心，以长浏高速、金洲大道为走向，分工特色鲜明、产业结构合理的工业经济走廊。二要推动工业自主创新，提高工业核心竞争力。充分发挥在长高等院校和科研院所在研究创新方面的优势，建立和完善合作机制，促进科技成果转化。加强创新平台建设，重点支持新建企业技术中心、重大技术创新项目、信息化建设示范企业、关键共性技术攻关，提高企业创新能力，大力发展创意产业，实现工业增长由投入拉动向创新驱动转轨。继续实施质量振兴战略，支持花炮工程技术中心建设，新增3个中国名牌产品和驰名商标，实现由产品竞争向品牌竞争转变。三要大力开展节能降耗，积极发展循环经济。创建全国节能型试点城市，加强工业和建筑领域的节能降耗，重点支持能源的合理利用和新能源、新技术的开发应用，节约集约利用土地。进一步完善节能工作机制，建立健全发展循环经济的机制保障，建立新引进企业项目能源消耗评价制度。开展“百家企业节能工程”，定期公布重点行业、重点企业的能耗和污染情况，并作为考核企业绩效、支持企业发展的重要依据。全面推行清洁生产，认真开展循环经济园区和企业试点工作。四要深化国有企业改革，切实增强发展活力。继续推进和全面完成市属国有企业改革，加快分离企业办社会职能，积极稳妥做好企业政策性破产和不良债务化解工作，解决好企业历史遗留问题。做好中央、省属企业改制后的交接工作和企业改制后社会职能移交社区管理工作。加快非公有制经济发展，鼓励民营资本和社会资本参与国企改革。加强企业家队伍建设，强化国有资产监管和经营业绩考核，防止国有资产流失，提高企业经营效益。

　　（二）突出发展现代农业，强力推进新农村建设。发展现代农业是新农村建设的首要问题。一要加快构建现代农业的产业体系。加强农业产业化基地建设，抓好四大优势产业带的提质增效，进一步优化农业结构，主要农产品优质率达到75%以上。加大农产品加工企业培育和支持力度，抓好红星实业300万头生猪屠宰加工、南山食品10万吨乳品加工、加加酱业技改扩建等重点项目。加快农产品加工园区发展，引导农业高科技企业和项目向园区集聚。大力发展农民专业合作组织，建立和完善现代农业市场体系，以产业和市场升级促进农业增效、农民增收，推动县域经济蓬勃发展。二要强化现代农业的科技和人才支撑。加快新型农业技术推广体系建设，加强先进适用技术推广。推进农业标准化和规模生产，完善动物防疫体系、农产品质量安全检验检测体系，着力培育品牌产业和产品。开展现代农业生产知识和技能培训，拓宽农村劳动力转移和劳务输出渠道，培养一批科技明白人、流通经纪人和致富带头人，以科技进步和劳务输出促进农民增收。三要提高现代农业的设施装备水平。加强农村规划、农田水利、农村公路、农网改造等建设，不断改善农村生产生活环境，提高农业综合生产能力。改善农机装备水平，加强先进适用农机具的示范推广，提高农业生产机械化作业水平。继续推进百村示范和千村整治工程，年内完成20%的新农村示范村建设主要任务。四要完善发展现代农业的保障机制。继续推进以乡镇机构、农村义务教育、县乡财政体制为重点的农村综合体制改革。积极推进水利投融资体制、水利工程管理体制和集体林权管理改革。逐步建立健全财政支农资金稳定增长机制，确保新增财力向农村倾斜。

　　（三）突出发展现代服务业，加快打造商贸大市、文化名城、旅游休闲之都。服务业是长沙的优势，要继续做强做优。一要大力发展现代新型服务业态。在改造提升商贸、餐饮、娱乐等传统服务产业、充分发挥既有优势的同时，要突出发展现代物流业，重点建设好金霞海关保税物流中心。大力发展电子商务、金融保险、专业代理、产权交易等现代生产性服务业和专业服务业，推进中央商务区建设，发展壮大楼宇经济和节会经济。大力推进大型连锁超市向农村扩张，拓宽消费领域和层次，继续保持和扩大商贸流通优势。二要大力发展文化产业。坚持历史文化名城保护与文化产业发展相结合，加速实现具有鲜明地域特色的长沙文化资源向优势文化产品的转化。提升歌厅和民间文化娱乐品质与服务水平，培育壮大新闻出版、影视传媒、卡通动漫、文博会展等八大优势文化产业，突出抓好动漫游戏产业振兴基地建设，支持宏梦、三辰等卡通争创全国创意产业优势品牌。三要大力发展旅游休闲产业。全面启动“中国最佳旅游城市” 创建工作，加快旅游交通、旅游产品和管理服务三大体系建设，逐步实现长沙由国内旅游向国际旅游目的地转变。全面提升和打造文化旅游、红色旅游和生态休闲旅游品牌，加大旅游市场开拓和营销力度，精心办好2007长沙国际娱乐旅游节和首届中国演出娱乐博览会暨歌厅文化节，努力将长沙建设成为全国知名的文化旅游休闲之都。

　　（四）突出推进区域性中心城市建设，增强城市集聚、辐射、带动能力。城市建设是经济社会发展水平的主要标志。一要科学规划和拓展城市发展空间。科学合理调整和完善城市规划布局，抓好重点地段城市设计，整体提升城市形象。以城乡一体化规划和交通融合为纽带，认真做好城乡结合部和长株潭结合部控制性详规编制工作，加速长沙县、望城县与主城区的融合，加快浏阳市、宁乡县与大城区的对接。推进长株潭的城际对接和深度融合，开办长株潭电视频道，增强长沙对“3＋5”城市群的辐射互动作用。提高卫星城镇和中心镇建设和管理水平。年内建成区面积扩大10平方公里，城市化水平提高2个百分点。二要不断完善和增强城市综合功能。完成城市建设重点工程项目101个，总投资119亿元。加快湘江路南北延伸工程、橘子洲生态风景区、三角洲现代新城、霞凝港三期工程、武广客运专线长沙段、火车南北货站迁建、引水及水质环境工程、中信新城、长沙电厂、黄花机场扩建等重大项目建设，抓紧做好轨道交通、湘江长沙综合枢纽工程项目报批工作。突出城市公交站场和停车场建设，加快污水处理厂建设和垃圾场改造工程进度。有序推进社区标准化和站厕建设，继续综合整治市容环境和推进旧城改造。以文化为灵魂，努力把长沙建设成为具有独特个性的历史文化名城与凸现鲜明时代特征的现代标志性建筑群落相交融、历史人文景观与自然生态景观相映衬的令人羡慕和向往的充满活力、美感、舒适感、环境优雅的最佳宜居城市。三要切实提高城市综合管理和服务水平。进一步完善城市管理体制，强化基层城管责任制和执法，推进城市管理向背街小巷和社区延伸。切实抓好市容环境卫生门前三包责任制的落实，建立健全全民参与城市管理的新机制。加强城市交通综合协调与管理，实行城区和城市出口电子全监控。认真实施城市亮化工程，精心打造区、街和行业亮化点，用生动、形象的文化创意构建点、面、线结合，空中与地面交相辉映的立体城市亮化景观。

　　（五）突出创新融资方式和机制，全方位、深层次扩大对外开放。扩大开放是实现长沙又好又快发展的强大动力。一要提升招商引资的层次和服务水平。突出引进世界500强和其他知名跨国公司等战略投资者，力争实现外资银行、保险公司来长设立分支机构零的突破。加强招商引资项目建设，构建立体招商网络，精心组织参加“澳大利亚投资贸易洽谈会”、“第二届中部博览会”等重大招商及经贸活动。完善项目督办机制，提高项目履约率。全年实际到位外资增长15%以上。二要创新和拓宽融资渠道。推进区域性金融中心建设，加快建设芙蓉中路金融街。扶助企业上市融资和发行债券，探索租赁融资、股份合作等融资方式，破解城市建设、产业发展、中小企业融资难问题。抓好二环线资产证券化工作，安排财政预算资金采购已建成的二环线工程。推进政府投资公司的改革重组，全面深化政府公共投资体制改革。做好扩大利用外资贷款和对外融资工作，引导和调整投资方向，力争工业和生产性服务业投资比重达到40%以上。三要努力扩大对外贸易与合作。巩固亚洲市场，拓展欧美非市场，优化出口商品和贸易结构，实现市归口管理企业进出口总额25亿美元以上。加强出口和外派劳务基地建设，支持对外投资和跨国经营，发展境外工程承包和劳务合作。深化与泛珠三角、中部地区的经贸合作，主动承接长三角产业转移，精心组织第四届泛珠三角经贸洽谈会、省会城市市长论坛、商会合作交流会和中国商标节。发挥长沙高新技术、文化、旅游、商贸、会展等优势，打造具有象征意义和强烈吸引力的长沙城市品牌。

　　（六）突出经济社会协调发展，努力建设和谐长沙。统筹经济社会发展是落实科学发展观的重要内容。一要努力实现充分就业，切实提高社会保障水平。统筹城乡就业，全面落实各项就业政策，进一步加强公共就业服务，多途径扩大就业、再就业，引导自主创业。大力发展职业技能培训，扩大农村劳动力转移培训“阳光工程”实施范围。努力提高社会保障覆盖面，重点抓好城镇居民医疗保险、被征地农民和农民工养老保险参保工作，做实养老保险个人帐户，启动养老、医疗、生育保险城区统筹。加快发展社会慈善事业。二要实施科教先导战略，加快社会事业全面发展。推进创新型城市建设，市本级科技三项经费增加到1亿元；大力普及科学技术，培育一批具有自主知识产权、高成长性的科技企业和产业；加大知识产权保护力度，创建国家知识产权示范城市。坚持教育优先发展，全面实施素质教育，促进义务教育均衡发展，推进普通中小学校标准化建设，大力发展职业技术教育，努力提高高等教育质量。进一步完善公共卫生体系，突出改善城乡基层医疗卫生机构的服务条件，提高新型农村合作医疗参保率和保障水平。推进文化体制改革，加快公益性文化设施和历史文化街巷建设，有序推进马王堆汉墓和铜官窑申遗，办好中国第13次历史文化名城年会和全国文化信息资源共享工程试点。加快农村广播电视“村村通工程”建设，完成城区有线数字电视的整体平移。加强全民健身运动，搬迁建设市体育馆，组团参加第六届全国城市运动会，推进健康型城市创建工作。加强人才工作，以培养和造就高层次和高技能人才为重点，带动人才队伍建设。坚持稳定低生育水平，统筹解决人口问题。切实抓好统计基层基础工作，认真搞好全国第二次农业普查。加强未成年人保护、教育工作和老龄工作，为青少年和老年人的学习、休息、娱乐等创造更好的条件和环境。三要建设生态、文明、平安、和谐长沙，全面提升城乡文明程度。严格国土资源管理，加强环境保护和生态建设，实行严格的环境准入标准，重点整治城区建设扬尘，积极防治农村面源污染，严格森林、湿地、水资源保护和管理，加强城市生态圈和生态主题园建设，争创全国环境保护模范城市。继续深入开展全国文明城市创建工作，加强以“八荣八耻”教育为主要内容的公民道德建设，提高市民综合素质。深化“平安长沙”创建工作，加强社会治安综合治理，切实维护国家安全和公共安全。加强民族团结，促进宗教和睦。进一步加强国防教育和国防后备力量建设，争创全国双拥模范城市。进一步畅通群众诉求渠道，加大信访事项督办力度，落实信访工作责任制。完善法律援助服务体系，维护社会贫弱群体的合法权益。继续实施食品、药品、农产品放心工程，建立质量安全适时监督发布和应急机制。进一步完善安全生产责任体系，突出重点行业和重点领域的安全专项整治，严防重特大安全事故发生。全面清理整治无证无照非法经营行为。加快社会信用征信体系建设步伐，推进诚信长沙建设。

　 三、建设务实、创新、廉洁的服务型政府

　　实现今年经济社会发展目标，必须切实加强政府自身建设，全面提高政府服务和管理经济社会发展的能力和水平。

　　（一）加强制度建设，推进政府管理创新。积极应对入世保护期结束带来的深层次影响，加快政府职能转变，推进电子政务建设，创新政府管理和服务。切实抓好公务员法实施，进一步规范公务员管理。加快建立和完善重大行政决策专家论证和评估制度、重大事项社会公示与听证制度、决策反馈纠偏机制和决策责任追究制度，建立政府及其部门自觉接受同级人大及其常委会的法律监督和政协的民主监督制度，完善政府及政府有关部门与民主党派、工商联对口联系制度，实现政府决策的民主化和科学化。深化行政审批制度改革，大力推行限时岗位办结制度和电子监察管理，减少事前审批，强化后续监管，建立行为更规范、运行更高效的行政审批运行机制，进一步降低行政成本，提高行政效率。强化行政监察和效能监察，加强对工程建设、土地出让、产权交易、商品采购等招投标的统一管理。大力推行政务公开，实行重大事项和公共管理事项新闻发布制度。进一步放松对具体经济事务的管制，降低准入门槛，鼓励全民创业，支持企业家、留学归国人员和务工返乡人员大胆创业，放心创业。对企业和个体工商户初次轻微违反有关规定实行教育和上门指导服务，着力营造法治、和谐、宽松的发展环境，使长沙成为最具创造活力和最适宜创新创业的城市。

　　（二）健全公共财政体系，切实关注民本民生。按照公共财政要求，切实把政府工作的着力点和财政的主要功能更多地放在提供公共产品与公共服务上，新增财力要进一步向基层和民本民生倾斜。通过加大投入和政策支持力度，不断完善农村基础设施和扶助山区贫困乡村脱困。要围绕解决人民群众的生活困难、看病难、上学难等实际问题，从今年起实现“四个全面”：即全面实行城乡义务教育“一费制”全免，全面实施城乡最低生活保障，全面实行城乡基本医疗保障，全面消除城镇零就业家庭。具体措施是：投入1.78亿元，从今年秋季起对城乡义务教育阶段学生实行“一费制”全免入学和补助农村贫困寄宿生生活费、资助特困家庭子女上大学；投入3.27亿元全面完善农村最低生活保障和提高城乡最低生活保障水平；投入2.6亿元建立健全城乡医疗卫生保障体系，支持每个乡（镇）建设一所中心卫生院、每个村建立一个卫生室、每个社区建立一个卫生服务中心，建立城市居民医疗保险制度；新增城镇就业8万人，切实解决城镇零就业家庭问题，新增转移农村劳动力5.5万人；投入3980万元完成农村五保户危房改造；投入1000万元完成40个建制镇和200个村的规划编制；投入2亿元建设改造干线公路和1053公里农村公路；加大对水利等基础设施建设支持力度，治理80座病险水库，建设40处安全饮水工程和1000公里标准渠道，改造中低产田8万亩，新建沼气池2万个；新建经济适用房50万平方米，建设廉租房1万平方米；投入1亿元完成100个社区的综合环境整治。真正让公共财政惠及全市人民，让全市人民共享改革发展成果。

　　（三）坚持从严治政，树立良好的政风。要切实增强廉洁和自律意识，认真执行党风廉政建设责任制和廉洁自律的各项规定，深入开展治理商业贿赂工作，严厉查处和坚决打击各种贪污腐败行为。要切实增强职责和服务意识，特别要加强对政府职能部门审批环节的监督，重点查处和解决行政审批中的不正之风和刁难卡压企业的行为，为企业发展壮大创造宽松的良好环境。要切实增强使命和责任意识，每一个公务员都要心怀中华振兴，情系百姓冷暖，坚持把实现长沙又好又快发展作为自己义不容辞的崇高使命，把解决人民群众的困难、疾苦作为自己不可推卸的重大责任。要经常深入基层调查研究，倾听人民群众的意见和呼声，以民忧为己忧、以民求为己求、以民乐为己乐、以民安为己安，一步一个脚印，一事一片真情地为维护、实现和发展好人民群众的根本利益披肝沥胆，在所不惜。要大力倡导求真务实，反对喜功浮躁，为想干事能干事的人创造干事的机会，让能干好事干大事的人有大显身手施展德才的舞台。要大力弘扬艰苦奋斗，反对铺张浪费，坚持领导带头、机关带头，全市政府机关要尽力节约办公经费，多为基层、为群众办实事、好事。要用实在的行动，过硬的作风，发展的成效，树立清正廉洁、勤政为民的良好政风，决不辜负全市人民的殷切期望！

　　各位代表，历史翻开了新的一页。保持和扩大长沙良好的发展态势，实现经济社会自然和谐发展，我们肩上的任务光荣而艰巨。让我们在市委的领导下，同心同德，奋发图强，披荆斩棘，阔步向前，为加快建设繁荣创新文明和谐的长沙，创造更加美好的生活，共同书写新时期的灿烂篇章！
